# Supplementary material for: Hurricanes and hashtags: Characterizing online collective attention for natural disasters
Source: PLoS One. 2021 May 26;16(5):e0251762. doi: 10.1371/journal.pone.0251762 (PMC8153433; doi:10.1371/journal.pone.0251762)
Supplement: S1 File — (PDF) [file pone.0251762.s001.pdf]

S1 File

1 Alternate Aggregate Attention Measures

While we chose to study the scaling of attention using a single restrictive hashtag or 2-gram for each storm, there are many hashtags that may be used to refer to any one storm. For instance the hashtags “#houstonstrong”, “#harvey”, and “#prayfortexas”

Mean Regression Parameters – Deaths

	Tropical Storms	Cat 1	Cat 2	Cat 3	Cat 4	Cat 5	All Hurricanes
a_{deaths}	0.26	0.61	0.41	0.70	1.43	1.39	1.16
a_0	-7.66	-6.60	-6.63	-6.23	-6.04	-6.92	-6.56

Mean Regression Parameters – Damages

	Tropical Storms	Cat 1	Cat 2	Cat 3	Cat 4	Cat 5	All Hurricanes
a_{damage}	0.13	0.28	0.23	0.35	0.47	0.46	0.31
a_0	-8.41	-8.28	-7.75	-8.19	-8.58	-8.20	-7.92

S1 **Table.** Mean Regression Parameters fit for storms of each category. See Fig 4 for full parameter distributions.

are all associated with the 2017 storm Hurricane Harvey. Some hashtags are even associated with multiple storms, such as the relief focused “`#hr4hr`” or the general “`#hurricane`”. Using our n-gram usage rate dataset, it is not possible to correctly attribute the attention share of general hashtags to a particular storm. However, concerns remain that our single hashtag could be too restrictive, and miss large amount of attention.

To confirm the validity of our chosen hashtag, we constructed a more comprehensive measure of hurricane attention. First, we searched for tweets in a 0.1% subsample of tweets containing the 2-gram “`hurricane *`” for each Hurricane in the study period within the 100 day period after each storm's formation date. Within these matching tweets, we counted every hashtag. The majority of these co-occurring hashtags are not specific to the storm, such as “`#news`”. We also remove the pattern “`#hurricane*`” to avoid biasing this alternate measurement meant to confirm our initial choice. To help identify closely related hashtags, we plotted the usage rate of up to the top 20 most frequency co-occurring hashtags. Hashtags with attention spikes around the storm's dates of activity and which were related to the storm in question were added to a list of relevant hashtags. This list will miss hashtags with such low usage rates that they do not appear in our 0.1% sample, but these hashtags should not considerably add to our aggregate measures of attention.

For each storm we compute two measurements of attention:

- Summed Related Hashtags Usage Rate—a sum of all associated hashtags' usage rate for 28 days after hurricane formation
- Maximum Related Hashtag Usage Rate—the sum the usage rate for the most used associated hashtag for 28 days.

The result is shown in S1 Fig. The most obvious outlier, Hurricane Bill from 2009, was a curiosity in the meteorological community as powerful Category 4 storm, but caused relatively little destruction remaining at sea. Clearly, the measures are highly correlated. We also calculated Spearman's Rho, to find the rank-order correlation between our Integrated Usage Rate, from the main study, and the Summed Related Hashtags Usage Rate. We found $\rho = 0.92$, confirming this strong association between different measures. This increases our confidence that our Integrated

2 Summary Tables for Regressions

Provided for the reader here are tables of summary statistics of the estimated parameters in the regression models in subsections Attention and Impact Regressions by

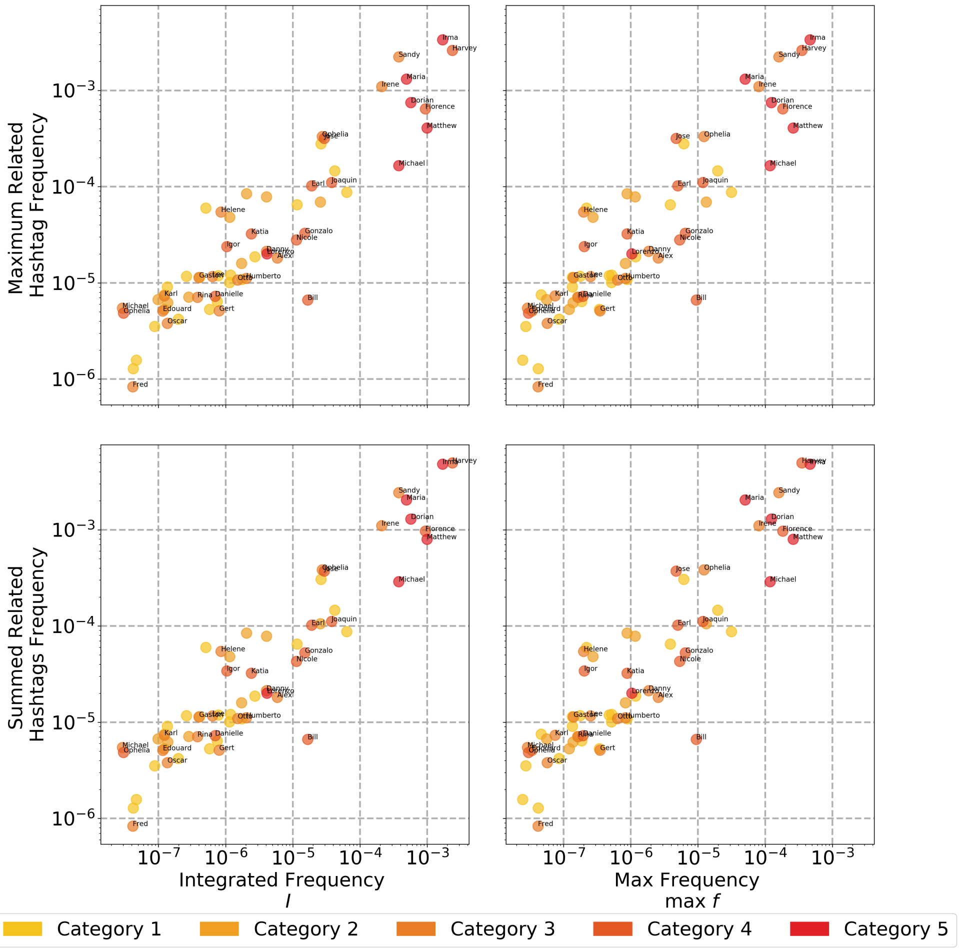

S1 Fig. Comparing Single Hashtag Attention Measures with Alternatives: Maximum Related Hashtag Usage Rate and Summed Related Hashtags Usage Rate

Category and Regression Models for Impacts, Impact Interactions and Hurricane Category.

a_0	a_{death}	a_{damage}
$\textbf{normal}(-8, 3)$	$\textbf{normal}(0, 1)$	$\textbf{normal}(0, 1)$

S2 **Table.** Priors for Regression 1

	mean	sd	mc_error	hpd_2.5	hpd_97.5	n_eff	Rhat
a_0	-7.57	0.52	0.01	-8.60	-6.56	4182	1.0
Deaths	0.49	0.16	0.00	0.16	0.80	4660	1.0
Damage	0.24	0.08	0.00	0.08	0.40	4108	1.0
sd	0.89	0.08	0.00	0.75	1.05	8449	1.0

S3 **Table.** Results for Regression 1

a_0	a_{death}	a_{damage}	$a_{\text{d,D}}$
**normal**$(-8, 3)$	**normal**$(0, 1)$	**normal**$(0, 1)$	**normal**$(0, 1)$

S4 **Table.** Priors for Regression 2

	mean	sd	mc_error	hpd_2.5	hpd_97.5	n_eff	Rhat
a_0	-7.58	0.51	0.01	-8.58	-6.58	8085	1.0
Deaths	0.05	0.34	0.00	-0.65	0.70	8326	1.0
Damage	0.22	0.08	0.00	0.06	0.38	8151	1.0
Interaction	0.06	0.04	0.00	-0.02	0.14	8676	1.0
sd	0.88	0.08	0.00	0.74	1.04	10843	1.0

S5 **Table.** Results for Regression 2

a_0	a_{death}	a_{damage}	$a_{\text{d}\times\text{D}}$	a_{C_i}
**normal**$(-8, 3)$	**normal**$(0, 1)$	**normal**$(0, 1)$	**normal**$(0, 1)$	**normal**$(0, 1)$

S6 **Table.** Priors for Regression 3

	mean	sd	mc_error	hpd_2.5	hpd_97.5	n_eff	Rhat
a_0	-7.64	0.51	0.01	-8.60	-6.60	9916	1.0
Deaths	0.09	0.36	0.00	-0.60	0.81	9892	1.0
Damage	0.20	0.08	0.00	0.05	0.35	10580	1.0
Interaction	0.05	0.04	0.00	-0.04	0.13	10424	1.0
Cat2	0.07	0.31	0.00	-0.55	0.66	15415	1.0
Cat3	0.21	0.26	0.00	-0.32	0.72	14877	1.0
Cat4	0.76	0.28	0.00	0.20	1.29	15063	1.0
Cat5	0.66	0.44	0.00	-0.17	1.57	13237	1.0
sd	0.84	0.08	0.00	0.70	1.00	14240	1.0

S7 **Table.** Results for Regression 3

3 2-gram Attention Proportion of "`hurricane`" Usage Rate

Examining the top 2-grams matching the pattern "**`hurricane *`**" in S3 Fig, we can get a sense of what are the top storms during the season, and how much attention is allocated to each at a given time. For English tweets, the first major spike of the 2017 hurricane season is surrounding Hurricane Harvey, though attention also spikes for Hurricane Katrina, in reference to the 2005 storm that affected a nearby region of the gulf coast. As attention begins to decay for Hurricane Harvey, a spike in usage for the 2-gram "**`hurricane relief`**" is observed, though it reaches only $f = 3 * 10^{-5}$. Next, attention turns to Hurricane Irma, which reaches the highest 2-gram usage rate of any hurricane in our dataset. Finally, one week after attention for Irma begins to decay, attention spikes for Hurricane Maria, though at a level noticeably lower than for Harvey or Irma.

We notice that during storm events the 2-gram usage rates for storms "**`hurricane *`**" is often between half or a fifth the usage rate of the 1-gram "**`hurricane`**", meaning that about one in every 5 times the name of the storm follows the word hurricane in English tweets during active storms.

In Spanish tweets the usage rates of "**`huracán harvey`**" only reach a maximum of around $f \sim 10^{-4}$, while "**`huracán irma`**" receives much more relative attention. "**`huracán marìa`**" receives about as much attention as Harvey, and also occupies a space similar to "**`hurricane maria`**" in English, around $f \sim 10^{-4}$.

	Integrated Frequency	Max Frequency	Deaths	Damage	Quantile 0.99	Quantile 0.9
2017 Harvey	2.3×10^{-3}	3.5×10^{-4}	107	1.2×10^{11}	126	14
2017 Maria	4.9×10^{-4}	5.0×10^{-5}	3057	9.1×10^{10}	363	166
2017 Irma	1.6×10^{-3}	4.6×10^{-4}	134	7.7×10^{10}	75	15
2012 Sandy	3.7×10^{-4}	1.5×10^{-4}	286	6.8×10^{10}	157	13
2018 Michael	3.7×10^{-4}	1.1×10^{-4}	72	2.5×10^{10}	201	13
2018 Florence	9.3×10^{-4}	1.8×10^{-4}	57	2.4×10^{10}	44	15
2016 Matthew	9.9×10^{-4}	2.6×10^{-4}	603	1.6×10^{10}	136	15
2011 Irene	2.0×10^{-4}	8.0×10^{-5}	58	1.4×10^{10}	14	8
2019 Dorian	5.7×10^{-4}	1.2×10^{-4}	70	4.6×10^{9}	36	12
2012 Isaac	2.6×10^{-5}	6.1×10^{-6}	41	3.1×10^{9}	192	97
2010 Alex	5.8×10^{-6}	2.5×10^{-6}	52	1.5×10^{9}	15	7
2017 Nate	6.3×10^{-5}	3.1×10^{-5}	48	7.8×10^{8}	8	5
2019 Barry	1.1×10^{-5}	3.8×10^{-6}	1	6.0×10^{8}	8	4
2016 Hermine	4.1×10^{-5}	1.9×10^{-5}	5	5.5×10^{8}	7	3
2019 Lorenzo	4.1×10^{-6}	1.0×10^{-6}	16	3.6×10^{8}	11	9
2014 Gonzalo	1.5×10^{-5}	6.4×10^{-6}	6	3.1×10^{8}	14	11
2015 Joaquin	3.7×10^{-5}	1.1×10^{-5}	34	2.0×10^{8}	11	5
2017 Ophelia	2.7×10^{-5}	1.2×10^{-5}	5	8.7×10^{7}	15	7
2009 Bill	1.6×10^{-5}	9.4×10^{-6}	2	4.6×10^{7}	11	7
2010 Earl	1.9×10^{-5}	4.9×10^{-6}	8	4.5×10^{7}	8	6
2014 Arthur	2.5×10^{-5}	1.3×10^{-5}	1	1.6×10^{7}	9	5
2016 Nicole	1.1×10^{-5}	5.3×10^{-6}	1	1.5×10^{7}	13	9
2017 Katia	4.0×10^{-6}	1.1×10^{-6}	3	3.2×10^{6}	7	4
2017 Jose	2.9×10^{-5}	4.7×10^{-6}	1	2.8×10^{6}	22	13
2014 Bertha	2.7×10^{-6}	1.1×10^{-6}	4	0.0	11	8
2015 Danny	4.0×10^{-6}	1.8×10^{-6}	0	NaN	6	3

S8 **Table.** The unnormalized values associated with radar plots in Results. Storms are colored by the maximum hurricane category from red as Category 5 to yellow as Category 1. As in the radar plots, storms are ordered by damage.

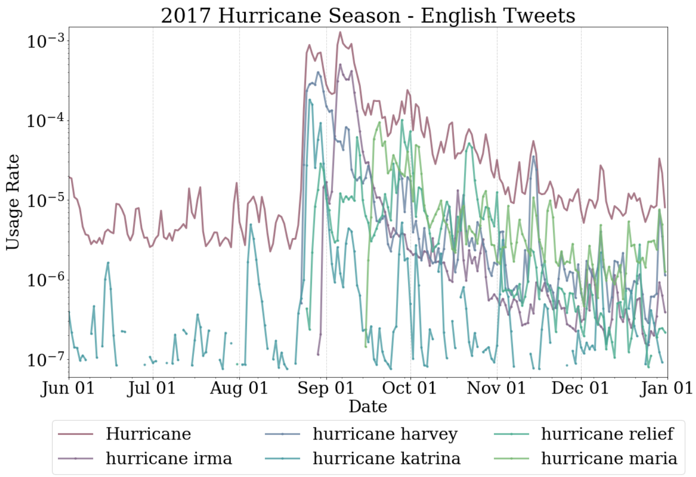

S2 Fig. Word usage rate proportions of “`hurricane *`” in English tweets

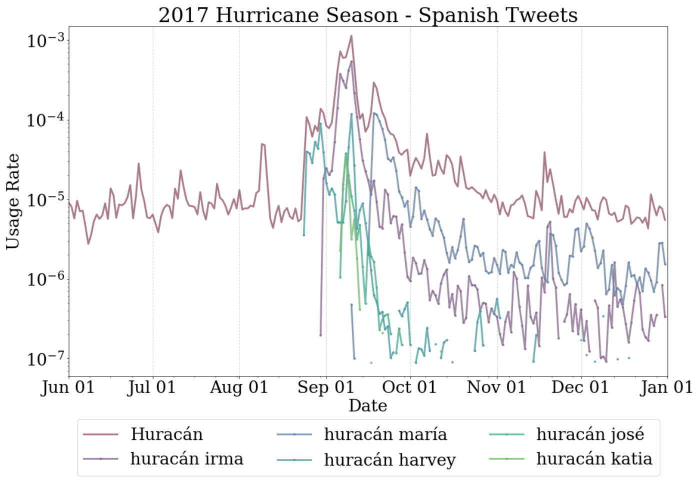

S3 Fig. Attention proportions of “`hurac´an *`” in Spanish. We can see that the word usage rate surrounding “`hurricane maria`” captures a similar amount of the total attention for the 1-gram hurricane as “`hurac´an mar´ıa`” captures. Additionally, Hurricane Harvey’s 2-gram usage rate is lower in Spanish than in English, while Hurricane Katrina is talked about considerably in English but does not rise about the 50000th most used 2-gram in Spanish. As always, usage rates are case-insensitive.

4 Bi-exponential Decays

To quantify the characteristic time scales of attention given to storms, we examined usage rates by fitting the bi-exponential model introduced by Candia et al. [33]. Not all storms receive enough attention, but 50 of 75 in the Atlantic basin recorded at least 6 days of consecutive 2-gram usage within the year of the hurricane, and these storms were had both their hashtag and 2-gram usage rate fit with the bi-exponential model of Candia et al.The model here assumes two populations, u and v, which become interested in a given event. Population u, comparable to the general population starts with a peak interest, and loses attention as $\frac{du}{dt} = -(p+r)u$. During every unit time pu attention is lost from the system and ru is transferred to population v. The dynamics of population v are as follows: $\frac{dv}{dt} = ru - qv$, so attention decays from v with rate q, but increases

proportionally to the total attention of population u. The final bi-exponential model is

$$S(t) = \frac{N}{p + r - q}[(p - q)e^{-(p+r)t} + re^{-qt}],$$

and we present the half-lives associated with this model as $\tau_1 = \frac{\ln(2)}{(p+r)}$ and $\tau_2 = \frac{\ln(2)}{q}$, which are the rates of decay from the two populations u and v. The distributions of τ_1 and τ_2 for both hashtag usage rates and 2-gram usage rates are shown in S4 Fig. The mean half-life for population u, the population with faster attention decay, is $\bar{\tau_1} = 1.3$ days for hashtags, and $\bar{\tau_1} = 1.1$ days for 2-grams. The decays for population v were not uni-modal, due to some storms regaining attention long after their initial impact, deviating from the model and receiving poor fits, and resulting in very large values of τ_2, but median values were approximately 24 days. All summary statistics are reported in S9 and S10 Tables. We speculate that for this model the population u is largely people effected by the storm, while population v is largely people writing about the storms or sharing i nformation about t he s torm response, eg, reporters and non-profit professionals. Further work could look to confirm who is behind the tweets.

The model we use makes an assumption that users tweeting with the hashtag do so within a role of one of two groups, where one group's attention is dependent on the attention of the other group. Other models, such as the one used by García-Gavilanes et al. citegarcia2016dynamics to study page views on Wikipedia, fit attention decays to a three-phase exponential model. Their model makes no explicit assumptions about subgroups of users and instead fits three sequential but separate exponential processes. This phased approach is useful for quantifying decay time series with dynamics that cannot be adequately described by a simple exponential, but assumes the three phases have unrelated decay rates. In Candia's model a smooth change in observed decay rate arises from the transfer of attention between two groups with different rates of attention loss. Further work can investigate whether this assumption of different groups is justified, but the model remains useful in our primary goal of summarizing the observed rates of change.

While we only found it necessary to use a bi-exponential model to adequately capture the decay dynamics, in general n-exponential decay models will assume a minimum of n decay rates, if there are no interaction terms. However, we are unable to observe n-grams with very low usage rates, so it is quite likely that a third regime exists with a decay rate operating at the year scale for historical storms. If this were the case a tri-exponential model would be appropriate, though unfortunately we would be unable to accurately fit all its parameters with our current data resolution.

The fitting procedure was to first find the maximum value of the usage rate for each storm, before fitting the above model to the decay of log usage rate after this maximum.

The resulting fits are shown in S7 and S8 Figs. The fits generally appear sensible, but there are sometimes issues for noisy time series, where the rate parameter r becomes very small, corresponding to a very long half-life, and misfitting the early decay. This occurs in the time series for Hurricane Florence. The distributions of Mean Squared Error (MSE) are shown in S6 Fig.

Looking at the decay half-lives in S9 Table we notice can see that most hurricane hashtags lose half their volume on the order of 1 or 2 days. The storms with relatively more attention on Twitter, Harvey, Irma, Matthew, and Sandy, all initially decay quickly, with a half-life on the order of a few days, but then have much longer decays associated with τ_2, on the order of a few weeks. There are some aberrations where the bi-exponential model does a poor job of explaining the data, such as for Hurricane Joaquin, where a fight between Governor Bobby Jindal and the Obama administration over the size of a recovery package spurred news stories and attention long after the initial activity associated with the storm itself. This leads to increases in hashtag usage

	Max Usage Rate	τ_1 [Days]	τ_2 [Days]	Season
#hurricanealex	2.5×10^{-6}	0.7	8.6	2010
#hurricanearthur	1.3×10^{-5}	0.9	190.3	2014
#hurricanebarry	3.8×10^{-6}	0.7	16.0	2019
#hurricanebertha	1.1×10^{-6}	0.6	6.9	2014
#hurricanebill	9.4×10^{-6}	0.2	693.1	2009
#hurricanechris	8.9×10^{-7}	0.6	693.1	2018
#hurricanecristobal	2.0×10^{-7}	2.0	6.9	2014
#hurricanedanielle	1.9×10^{-7}	0.7	693.1	2010
#hurricanedanny	1.8×10^{-6}	0.7	6.9	2015
#hurricanedorian	1.2×10^{-4}	1.6	8.8	2019
#hurricaneearl	5.0×10^{-6}	0.4	6.9	2010
#hurricaneflorence	1.8×10^{-4}	2.8	323.3	2018
#hurricanegert	3.6×10^{-7}	0.4	6.9	2017
#hurricanegonzalo	6.4×10^{-6}	0.9	693.1	2014
#hurricaneharvey	3.5×10^{-4}	2.5	30.6	2017
#hurricanehermine	1.9×10^{-5}	0.8	15.9	2016
#hurricaneida	8.3×10^{-7}	0.8	9.7	2009
#hurricaneigor	2.2×10^{-7}	1.1	693.1	2010
#hurricaneirene	8.0×10^{-5}	0.7	26.5	2011
#hurricaneirma	4.6×10^{-4}	1.0	20.0	2017
#hurricaneisaac	6.1×10^{-6}	0.7	693.1	2012
#hurricanejoaquin	1.1×10^{-5}	1.2	57.7	2015
#hurricanejose	4.7×10^{-6}	2.0	23.1	2017
#hurricanekarl	7.4×10^{-8}	0.6	68.9	2010
#hurricanekatia	8.7×10^{-7}	0.2	6.9	2011
#hurricanelorenzo	1.0×10^{-6}	1.3	64.2	2019
#hurricanemaria	5.0×10^{-5}	4.1	43.4	2017
#hurricanematthew	2.6×10^{-4}	1.4	27.4	2016
#hurricanemichael	1.1×10^{-4}	1.8	20.2	2018
#hurricanenate	3.1×10^{-5}	0.5	10.6	2017
#hurricanenicole	5.3×10^{-6}	0.6	6.9	2016
#hurricaneophelia	1.2×10^{-5}	0.3	6.9	2017
#hurricanesandy	1.5×10^{-4}	1.1	23.0	2012
#hurricanetomas	3.0×10^{-7}	0.9	6.9	2010

S9 **Table.** Fitted half-lives τ_1 and τ_2 for all storms with at least 10 days of observed hashtag usage.

	Max Usage Rate	τ_1 [Days]	τ_2 [Days]	Season
Hurricane Alex	4.1×10^{-5}	0.8	9.3	2010
Hurricane Arthur	2.8×10^{-5}	1.0	693.1	2014
Hurricane Barry	8.9×10^{-6}	0.6	6.9	2019
Hurricane Bertha	8.2×10^{-6}	0.4	693.1	2014
Hurricane Bill	8.2×10^{-5}	0.8	9.7	2009
Hurricane Chris	3.0×10^{-5}	0.6	693.1	2018
Hurricane Cristobal	1.9×10^{-6}	1.5	693.1	2014
Hurricane Danielle	1.0×10^{-5}	0.9	7.1	2010
Hurricane Danny	7.6×10^{-6}	0.6	693.1	2015
Hurricane Dorian	1.1×10^{-4}	2.6	18.2	2019
Hurricane Earl	1.7×10^{-4}	1.2	9.5	2010
Hurricane Florence	1.3×10^{-4}	3.5	37.1	2018
Hurricane Gert	1.0×10^{-6}	2.1	321.9	2017
Hurricane Gonzalo	1.4×10^{-5}	1.7	693.1	2014
Hurricane Harvey	4.0×10^{-4}	2.9	29.3	2017
Hurricane Hermine	2.0×10^{-5}	0.4	6.9	2016
Hurricane Ida	4.5×10^{-5}	0.7	17.1	2009
Hurricane Igor	1.1×10^{-5}	1.0	25.2	2010
Hurricane Irene	3.3×10^{-4}	1.2	21.8	2011
Hurricane Irma	5.0×10^{-4}	2.3	24.1	2017
Hurricane Isaac	3.8×10^{-5}	1.6	21.1	2012
Hurricane Joaquin	4.4×10^{-5}	1.2	144.5	2015
Hurricane Jose	2.4×10^{-5}	1.3	7.1	2017
Hurricane Karl	1.6×10^{-5}	0.3	6.9	2010
Hurricane Katia	9.3×10^{-6}	2.1	7.4	2011
Hurricane Lorenzo	2.7×10^{-6}	1.7	8.1	2019
Hurricane Maria	1.1×10^{-4}	0.7	6.9	2017
Hurricane Matthew	2.9×10^{-4}	1.7	22.4	2016
Hurricane Michael	9.3×10^{-5}	2.5	27.2	2018
Hurricane Nate	3.5×10^{-5}	0.5	693.1	2017
Hurricane Nicole	1.2×10^{-5}	0.3	6.9	2016
Hurricane Ophelia	1.9×10^{-5}	0.5	6.9	2017
Hurricane Sandy	5.3×10^{-4}	2.1	28.5	2012
Hurricane Tomas	1.4×10^{-5}	0.9	6.9	2010

S10 **Table.** Fitted half-lives τ_1 and τ_2 for all storms with at least 10 days of observed 2-gram usage.

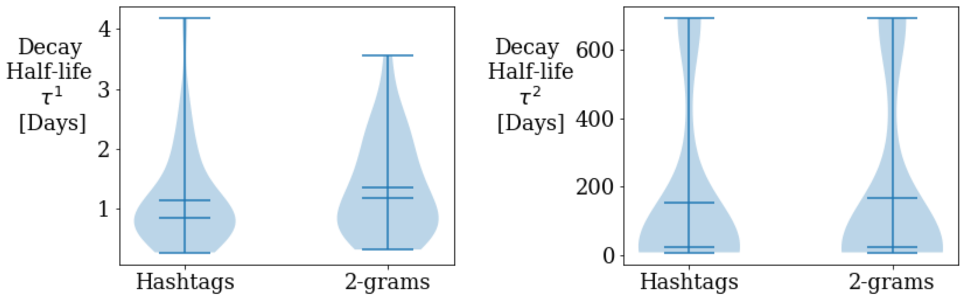

S4 Fig. Bi-exponential Hurricane decay half-lives: Distributions of fitted half-lives for the populations u and v. The mean half-lives for $\tau_1 = 1.3$ days and $\tau_2 = 156$ days for hashtags and $\tau_1 = 1.1$ days and $\tau_2 = 241$ days for 2-grams. For τ_2 the median half-lives are also interesting since we suspect the longest half-lives are due to poor fits. For hashtags $\tau_2 = 23$ days, and for 2-grams $\tau_2 = 24$ days.

rate, and thus negative half-lives. The longest half-life is associated with Hurricane Maria, τ_2 was approximately twice as long as the next largest hurricane. The extended crisis in Puerto Rico caused by Maria may be a reason this exceedingly long lifetime, even though the initial attention received by the hashtag was less than storms of comparable strength.

We also fit a simple exponential model $S(t) = Ne^{-pt}$. For high attention storms for which we have more than a week of data, this model is unable to capture decays occurring on different time scales, and thus has poor fits. For smaller storms for which attention is lower than the resolution of our data set, the exponential model is perhaps more appropriate. A distribution of half-lives for hashtags and 2-grams is shown in S5 Fig. While for larger storms, the fits did not capture the changing rates of attention decay, it was adequate for smaller storms that decay quickly below our instrument's resolution. However, for storms for which we have data for an extended decay, the bi-exponential model is more appropriate.

5 Hurricane Attention Maps

The remaining Hurricane Attention Map and time series from 2009 to 2018 are presented for the reader's perusal. Only storms reaching at least Category 2 are shown, and Seasons 2013 and 2014 are omitted. Earlier storms in our dataset mostly did not make landfall, and thus appear to receive relatively little attention. The scale of attention on the maps is held constant between years.

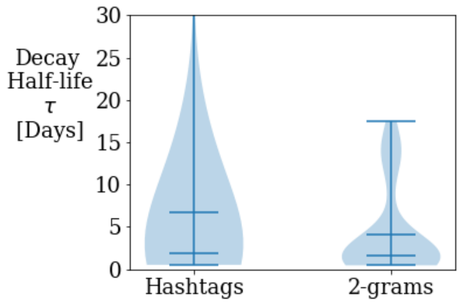

S5 Fig. **Simple Exponential Hurricane decay half-lives:** Distributions of fitted half-lives for a single population. The median half-lives for $\tau = 5.3$ days a for hashtags and $\tau = 5.2$ days for 2-grams. The simple exponential model fails to explain the break in attention decay for larger storms, receiving more attention. The bi-modal distribution of half-lives for 2-grams suggests that there are two categories of storms, ones with larger half-lives have more data, and thus the longer decay increases the fitted half-life. Meanwhile, smaller storms receive so little attention, that we don't measure any after a week or so, leading to a much smaller half-live, which corresponds to τ_1 in our bi-exponential fit.

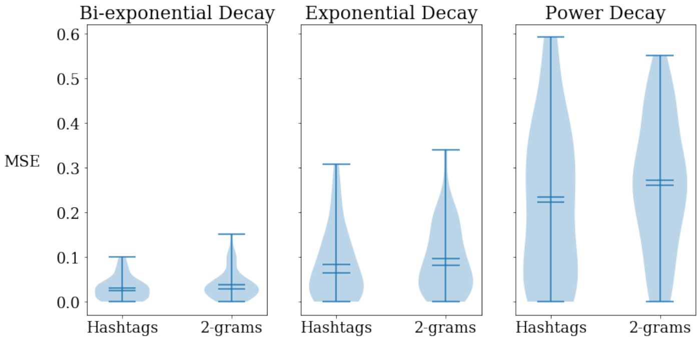

S6 Fig. Decay Model Comparison: Distributions of Mean Squared Error (MSE). The bi-exponential model has the lowest average MSE, followed by the simple exponential decay. The power law decay fails to capture the dynamics of attention decay, when the fit is compared to the data visually, and is reflected in the higher average MSE.

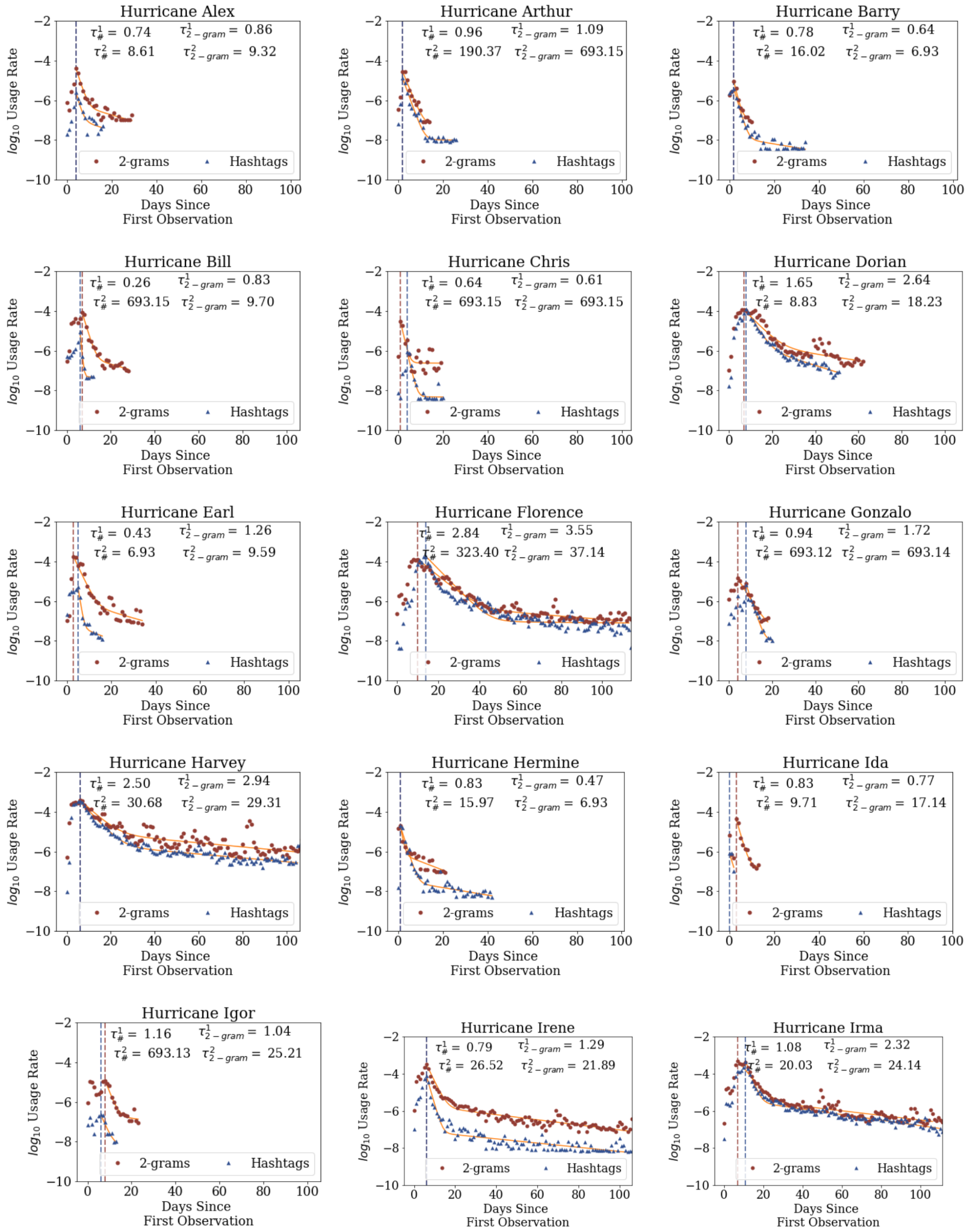

S7 Fig. Hurricane bi-exponential decay fits for hashtag usage rates and 2-gram usage rates for "`hurricane *`"

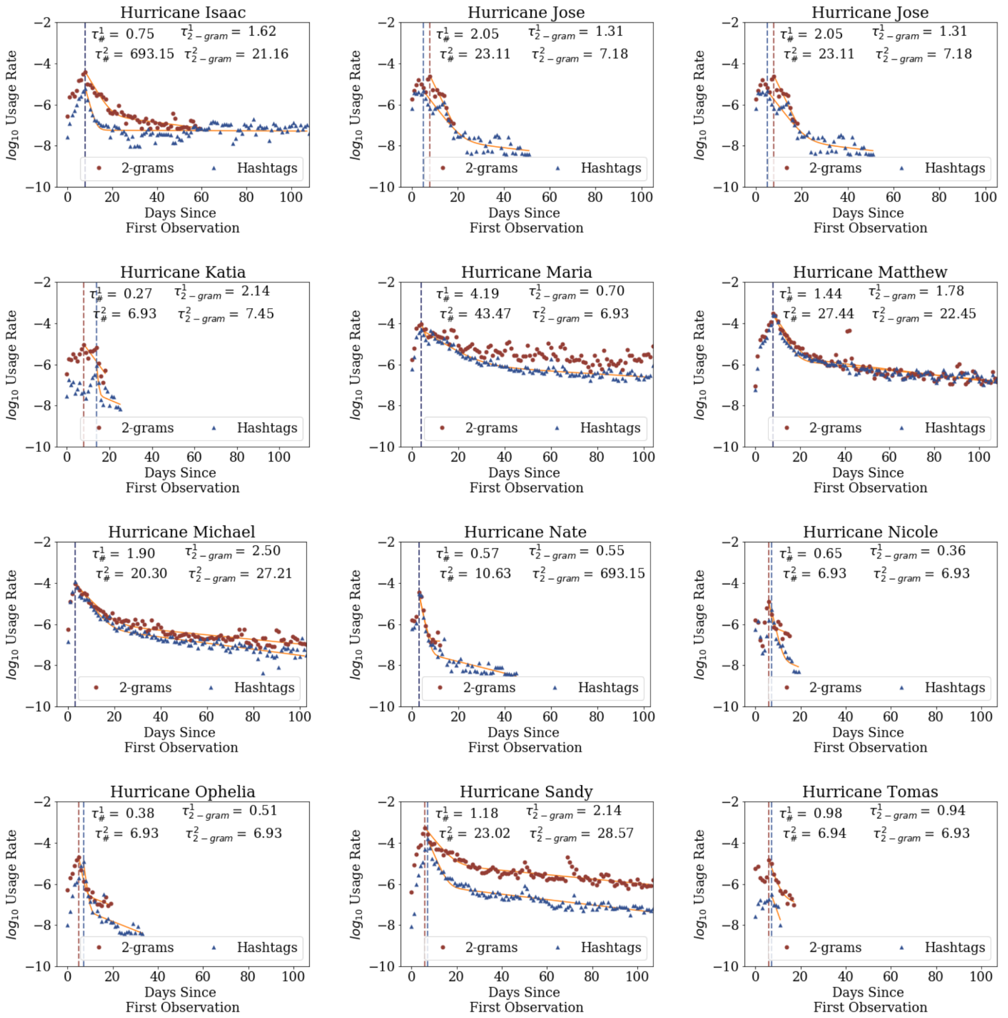

S8 Fig. Hurricane decays fits for all hurricanes for which we have at least 10 days of 2-gram usage rate data. Fits are performed for the function $y = \frac{N}{p+r-q}[(p-q)e^{-(p+r)t} + re^{-qt}]$, a simple two population decay model as proposed by Candia et al. [33]. Here p would be interpreted as rate of decay from population 1, r would be the transfer rate from population 1 to population 2, and r would be the rate of decay from population 2. Population 1 might be thought of as bystanders with a shorter attention span, while population two are those living with the ramifications, or working on the recovery who lose attention more slowly. Reported on the graph are the half lives associated with fitting this model for both the hashtag usage rate and 2-gram usage rate, $\tau_1 = \frac{\ln 2}{p+r}$ and $\tau_2 = \frac{\ln 2}{q}$

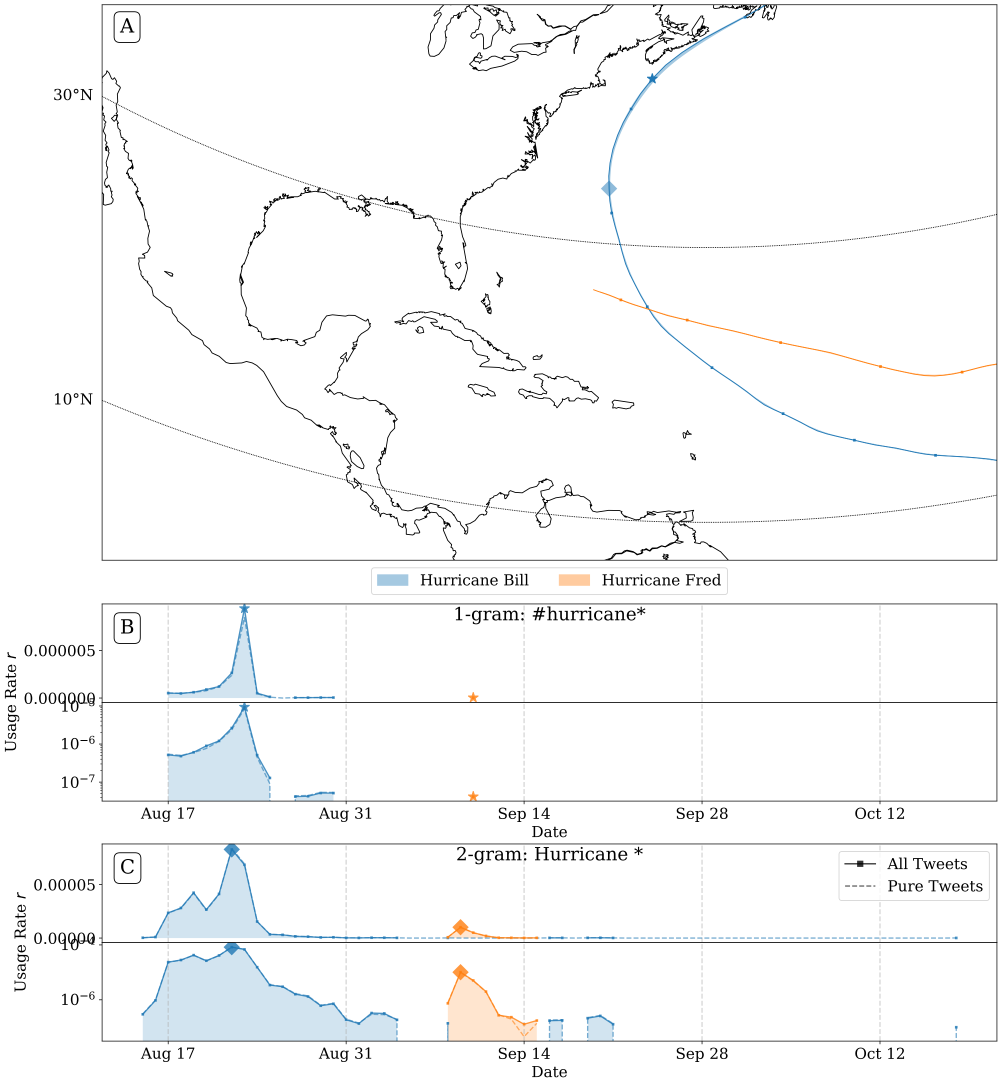

S9 Fig. Hurricane Attention Map and time series for 2009

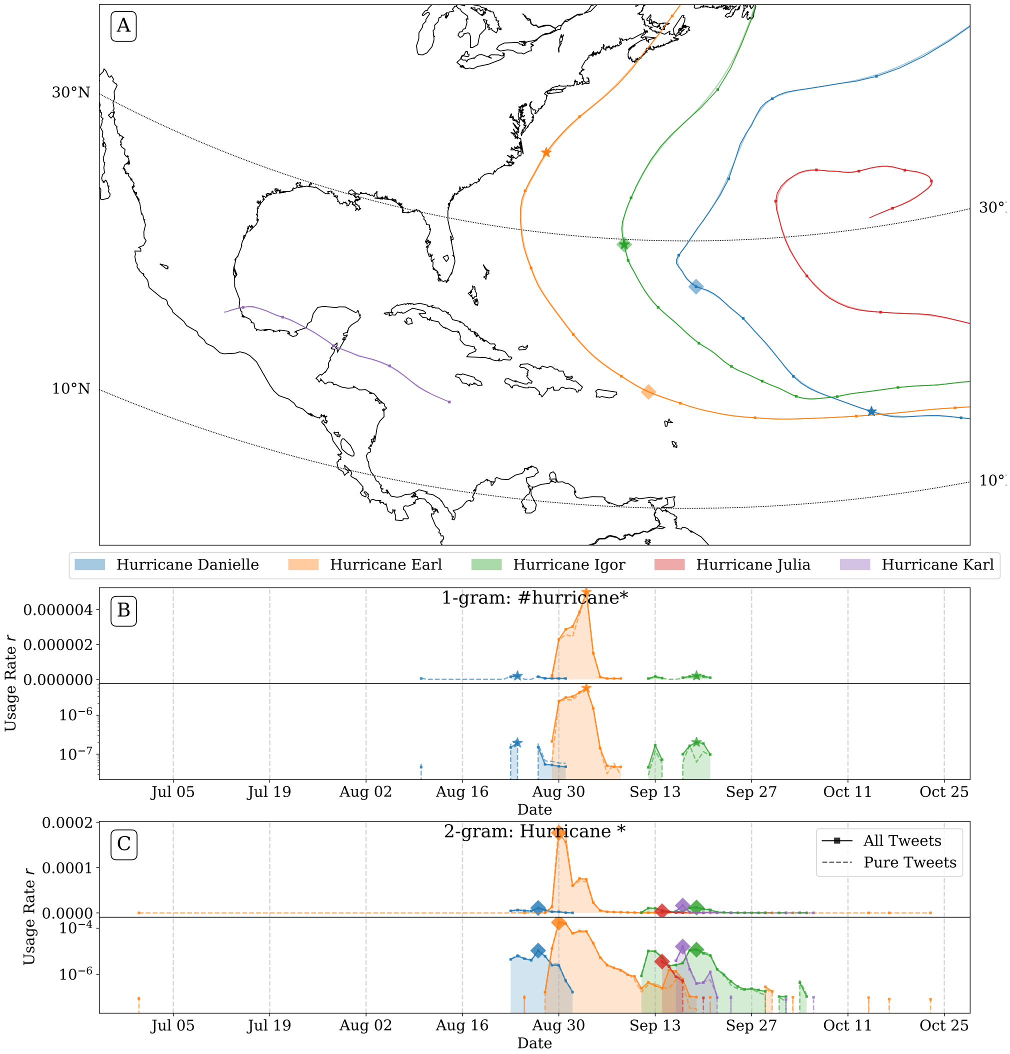

S10 Fig. Hurricane Attention Map and time series for 2010

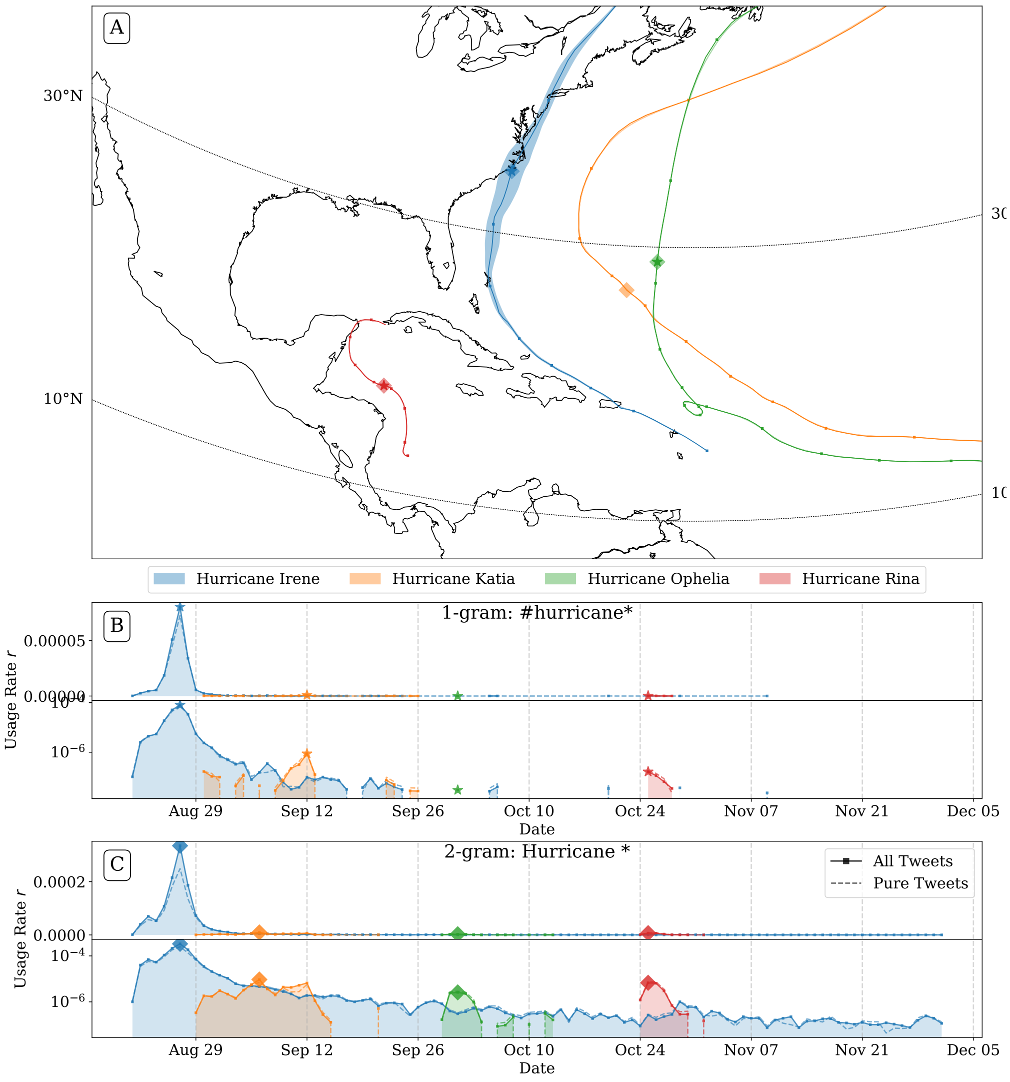

S11 Fig. Hurricane Attention Map and time series for 2011

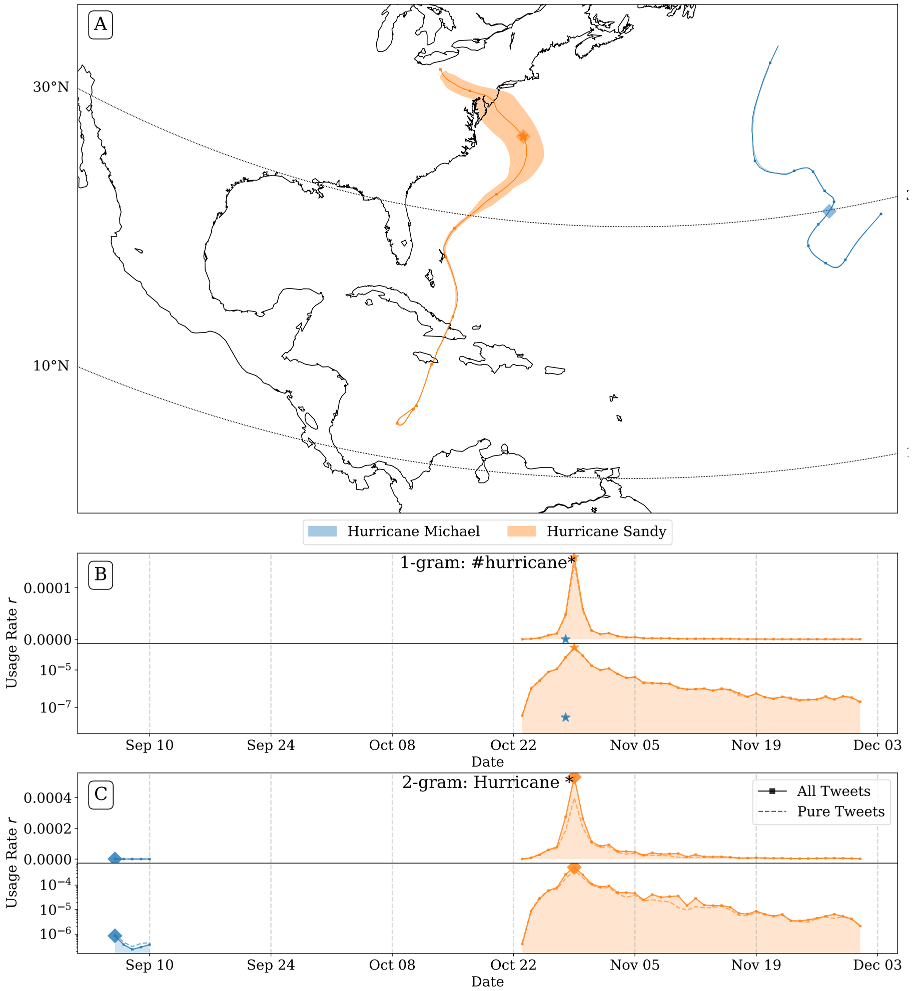

S12 Fig. Hurricane Attention Map and time series for 2012

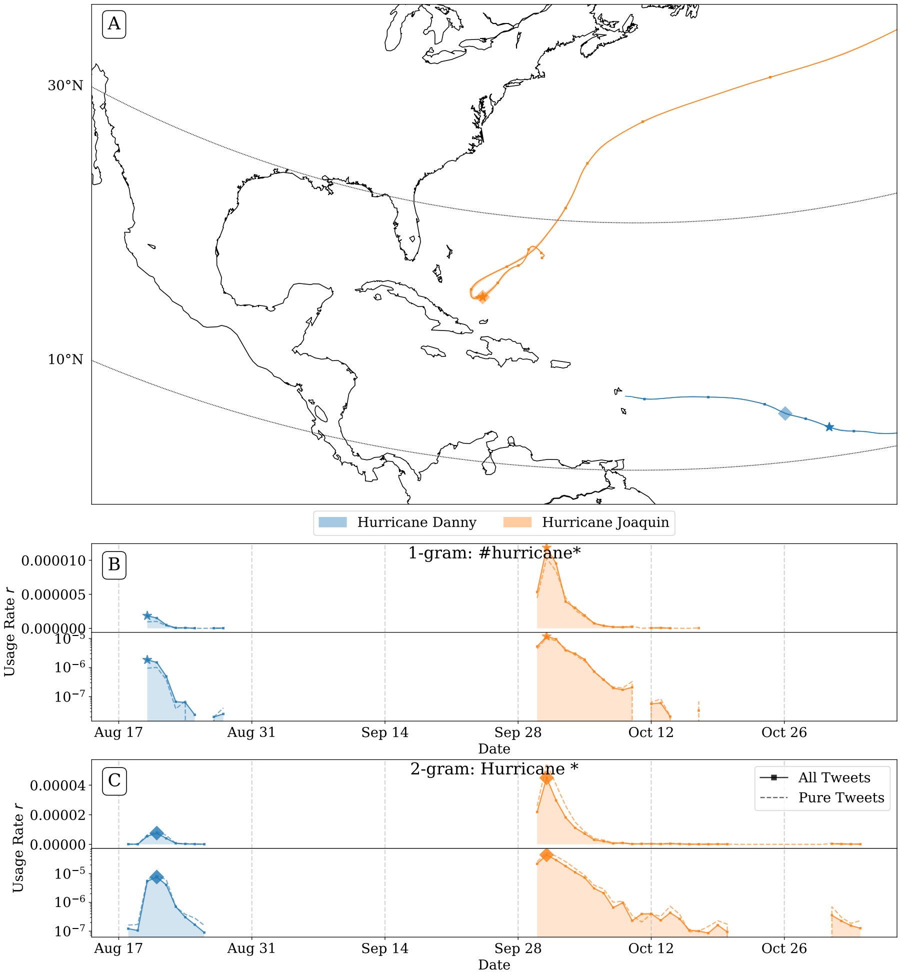

S13 Fig. Hurricane Attention Map and time series for 2015

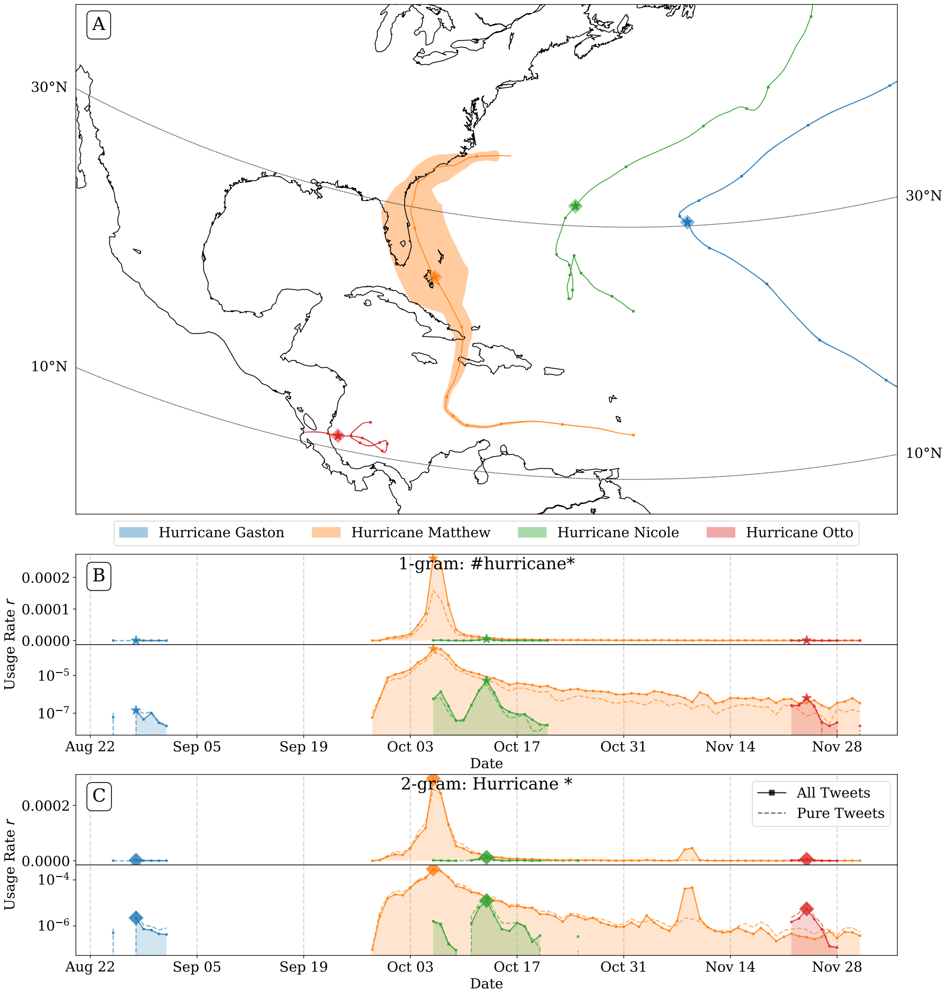

S14 Fig. Hurricane Attention Map and time series for 2016

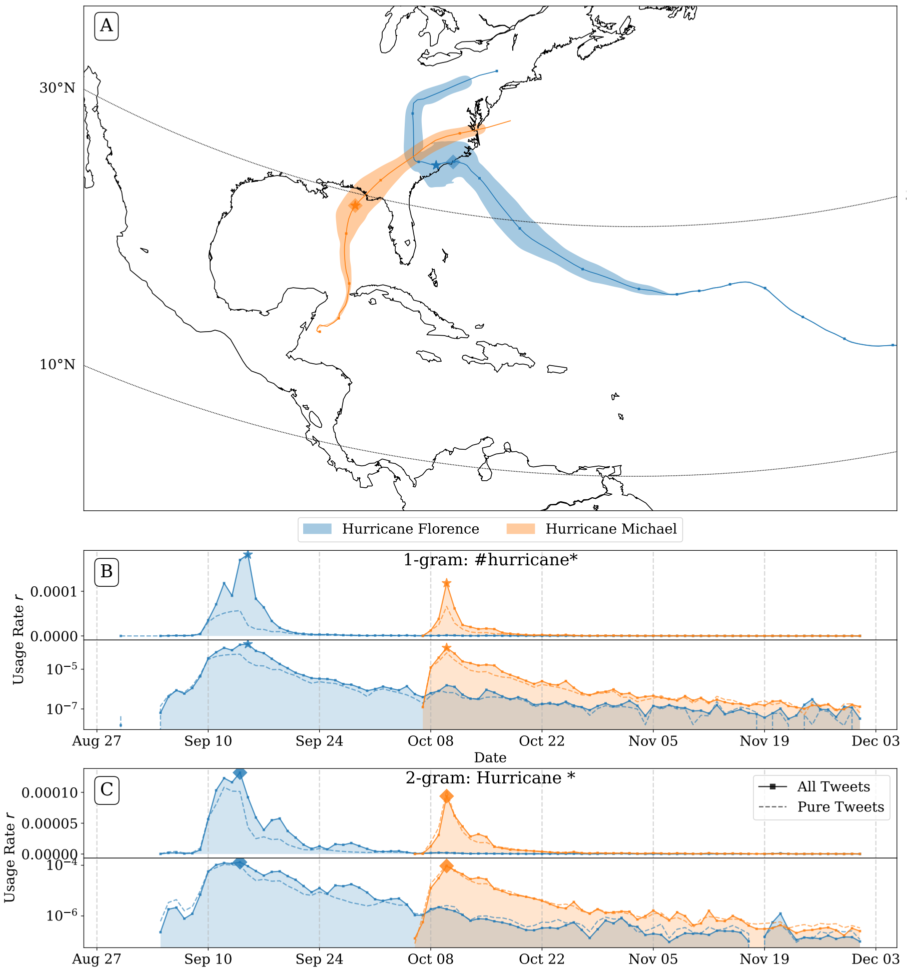

S15 **Fig. Hurricane Attention Map and time series for 2018**
