# Supplementary material for: Hurricanes and hashtags: Characterizing online collective attention for natural disasters
Source: PLoS One. 2021 May 26;16(5):e0251762. doi: 10.1371/journal.pone.0251762 (PMC8153433; doi:10.1371/journal.pone.0251762)
Supplement: Attachment — Submitted filename: Hurricanes_Response_to_reviewers.pdf [file pone.0251762.s002.pdf]

Response to Reviewers

To the editors and reviewers,

Thank you for the thorough and thoughtful reviews of our manuscript. We have carefully read and considered the suggestions therein and have made substantial revisions to our paper, which we now feel has been improved greatly. We discuss further our response to individual concerns below.

Response to Academic Editor

Comment: *While all reviewers agreed that the analysis is mostly sound and the data supports the conclusion, there were several comments and questions about the soundness of the study, particularly regarding the narrow criteria for identifying related tweets (Reviewer 1) and justification for the choice of "bi-exponential" decay model (Reviewer 3). In addition, reviewers pointed out many errors and inconsistencies in the current version of the manuscript.*

Response: We thank the editor and reviewers for these suggestions, and have made some substantial revisions to address the stated concerns.

To address our narrow criteria for identifying related tweets, we created a parallel measure of attention relating to each hurricane with a much broader criteria. We identify related hashtags that co-occur with each storm's 2-gram, e.g. "Hurricane Maria," and compute new attention measures based on this much broader criteria. We find that the new measure is highly correlated with our initial narrow criteria measurement.

To further justify our choice of our bi-exponential decay model, we added a section explicitly explaining the assumptions, as well as comparing these assumptions to those of alternative models in the literature.

Additionally we have corrected the errors and inconsistencies pointed out by the reviewers.

Comment: *Finally, all reviewers pointed out lack of the data/code sharing. Because it is possible to share tweet IDs under the Twitter's Terms of Service and because sharing tweet IDs as a dataset is routinely done in many other research papers, I would strongly urge the authors to share their code and data.*

Response: We have now provided both data and code to reproduce major results on our project's public GitHub repo:

https://github.com/mvarnold/Hurricane-Data-Repo

Comment: *Finally, all reviewers pointed out lack of the data/code sharing. Because it is possible to share tweet IDs under the Twitter's Terms of Service and because sharing tweet IDs as a dataset is routinely done in many other research papers, I would strongly urge the authors to share their code and data.*

Response to Reviewer #1

Comment: *One limitation is that looking only at #hurricane* and "hurricane *" could miss a lot of information. Take the example of Hurricane Maria. First, since the disaster relief response was botched, many people might have quit using the hurricane hashtag to focus on more topical elements (even just #puertoricostrong), although they were still tweeting about the effects of the hurricane that, in other cases, might have warranted the use of the "official" hashtag.*

Response: We agree that looking at only #hurricane* and "hurricane *" do miss many topical tweets, even a majority of the tweets associated with the storms. Certainly storms are frequently referred to only by their name, e.g. "Maria left a Puerto Rico without power", or just generically as the hurricane or storm. These less formal references overlap with common given names, or other weather events, introducing more uncertainty to our measure. However, we feel reasonably confident that tweets containing the hashtag and 2-gram form during the year of the storm are overwhelmingly referring to the weather event under study.

To address the concerns of the reviewer we have constructed a new broader measure of attention, noted its existence in the paper, and detailed the method of computation in the Supplemental Information. In short we identified more related hashtags for each storm by look at hashtags occurring with the 2-gram "hurricane *" in a large 0.1% subsample of tweets, and find a high association between these new measures of attention, and our original measures (Spearman's Rho = 0.92). We also intentionally withheld hashtags matching the pattern "#hurricane*" to avoid biasing these measures towards our initial measurement choice.

Comment: *Second, a lot of activity might simply have ended up under #huracanmaria and #huracánmaria, which are two distinct but still very popular hashtags. Note that the authors thought about this, but they looked for the hashtag with the wrong spelling (at least according to the caption of Fig 7 and related appendix discussion): "huracàn" is not Spanish, "huracán" is the correct spelling and it is by far more popular. Regardless whether they got the accent right, both hastags are however eclipsed in usage by the simple (wrong) spelling "huracan". Since there are only 75 hurricanes in the dataset, it is not unreasonable to ask for more due diligence and trying to include related hashtags for them.*

Response: We thank the reviewer for pointing out this mistake with accents. Fortunately, this is only a typesetting mistake, which has been corrected. The correct Spanish form, "huracán", was searched for initially as is indicated in the relevant plot's legend.

Comment: *Authors claim that "damages could act as a priming factor for human attention, in part explaining why deadly disasters in capital-poor countries often receive less attention than when similarly deadly storms occur in wealthy areas". This is a bit of a stretch. An alternative hypothesis is that people tweet what affects them. If there are few material damages and lots of deaths, it might be that the hurricane hit a place with few Twitter users. Such a sophisticated explanations might also not be needed at all, since the "capital-poor" examples in the data are*

Mexico and Puerto Rico, which are Spanish speaking, while the vast majority of the analysis was made on English-speaking tweets.

Response: We thank the reviewer for this comment, and wholly agree with their analysis. Thus, we have removed this claim, and have added language acknowledging the uneven distribution of Twitter users, and our dataset's limitation of lacking Spanish language tweets.

Comment: *The authors are not very good at consistently reporting information throughout the paper. For instance, they report on page 8 that there are "only seven" storms of category 5, but then on page 9 they say that, in the same category, "n = 6". In the conclusion, they talk about 27-fold and 3-fold increase in attention with a 10-fold increase in deaths depending on the strength of the storm. However, the 27-fold figure is from the caption of Fig 3, which associates only a 2-fold (not 3-fold) increase for weaker storms. The 3-fold figure is from the last paragraph of Regression 1 (page 10), which is a different model, as far as I can understand. This confuses me and I have to say that, to be honest, following what's going on is really hard. Perhaps there should be a recap table somewhere?*

Response: In response to the reviewer's comments, we added a recap table (now Table 1) to summarize the interpreted regression coefficients. In doing so, we found minor inconsistencies between the fitted regression parameters, caused by the stochasticity in the bayesian fitting process, where previously we did not specify the random seed. We have now done so, and have updated the relevant results to this reproducible fit.

Additionally, we have corrected the error stating there were seven category five storms to the correct number.

Comment: *Some of the data is publicly available, but not all of Twitter data is. There is no code release to verify the results.*

Response: While we are not releasing tweet ids, we have subsequently published all intermediate data used in the study on our GitHub repo. Our starting point for this work was not tweets, but rather the usage rate time series.

We have a publicly released API for our general Storywrangler usage rate time series dataset. The attention time series dataset used in this study was from a 0.1 Version of Storywrangler focused only on English with higher resolution.

Additionally we have added the code to reproduce the major results to the public Github repo.

Comment: *- Last paragraph of the introduction has broken references. There are more broken references in the paper (e.g. in page 7), which makes it hard to follow.*
- When authors refer to Fig 6 to compare English and Spanish hashtags, they should refer to both Figs 6 and 7, since Fig 6 only has English info. Also, why are these references before referring Figs 2 to 5? Perhaps author should renumber their figures to take into account that some are in appendices.
- Some typos (e.g. "which we be referred to").
- Table 8 is a bit pointless since it is never discussed nor referenced in the text. It seems that is is

currently half-cropped out from the paper. It should also report the category of the hurricane (which is important so that we could see whether there are Cat 1 hurricanes which came close to inflict damages comparable to Cat 5 ones).
- Also Table 9 is never referenced, I think.

Response: In response to the reviewer suggestions we have fixed the noted broken references, added missing references, and added a "S" prefix for figures in the Supplementary Materials.

We have added explicit references to the tables in the main body of the text were they were lacking. We have also color coded what was Table 8 (now Table 9) by category.

We thank the reviewer for their comprehensive suggestions.

Response to Reviewer #2

Comment: *There is no description on how to make the dataset publicly available. Even though authors cannot do so as Twitter dataset is commercial, the author should state that.*

Response: Though the dataset of tweets is not publicly available, the attention usage rate dataset is provided on our project's Github page.

Comment: *Authors investigated the relations between the size/impact of Hurricane and the attention paid by the users in Twitter. I firstly want to argue if the novelty and the implications from the finding is enough to be published. To give implications on their findings, authors said that "... it could be advantageous for NGOs to prospect for financial contributions while collective attention is focused most strongly on a storm. It is also possible that the speed and scale of governmental relief programs are influenced by popular attention paid to storms, and previous work has shown that relief has been inequitable in the past". However, I think it is quite intuitive for NGOs and the government department, so there may not provide much implications to them. In line with that, I suspect the contribution in this paper is enough to be published.*

Response: We agree with the reviewer that the scaling of attention with Hurricane results may provide little useful information to NGOs. What may be useful is understanding the dynamics of that attention: exponential type decays with very short half-lives, meaning a window may exist for very efficient donation solicitation. We have added language to emphasize that the potentially useful information here is the timing.

Comment: *There are a lot of errors in the manuscript. In particular, all the section numbers are represented as ??, which seems to be made by Latex error. This makes me feel that authors have not fully revised their submission, so the submission should be revised and polished before being published.*

Response: The latex errors in the manuscript have been corrected.

Response to Reviewer #3

Comment: *The paper reports on an interesting question that is how the public attention is driven towards natural disaster. The study is limited to Twitter that is a weakness, however the authors provide a good discussion on the limitations of their approach. The analysis are straightforward and easy to follow. Would be good if more information is given on the hurricane categorisation for the authors unfamiliar with the topic.*

Response: We thank the reviewer for their comments and suggestion. We have added language in the Hurricane Category Regression section to give readers background on the Saffir-Simpson wind scale as well as context for why a study of collective attention might focus on this measure.

Comment: *I would like to see more theoretical justification for the choice of "bi-exponential" decay model. Particulalry I'd like to see how these assumptions compare to what is done in García-Gavilanes, R., Tsvetkova, M., & Yasseri, T. (2016). Dynamics and biases of online attention: the case of aircraft crashes. Royal Society open science, 3(10), 160460, where a 3-phase decay is modelled.*

Response: We have expanded the discussion around the choice of the bi-exponential model, and compared the assumptions in it to the reviewer's suggested model. In short, the bi-exponential model explains different regimes in the attention decay rate through the interaction of two groups with differing decay rates, while the model of García-Gavilanes assumes each of the three phases are separate simple exponential decays. We also note that our data resolution floor likely prevents us from observing a distinct third decay regime, such as that observed in wikipedia page views, but if we had better resolution we would might have to expand our model to a tri-exponential generalization to adequately capture the decay dynamics.

Comment: *I would also like to see a discussion on the interaction between different events, could some public attention be driven and triggered by other (similar) events as it's suggested in García-Gavilanes, R., Mollgaard, A., Tsvetkova, M., & Yasseri, T. (2017). The memory remains: Understanding collective memory in the digital age. Science advances, 3(4)*

Response: We agree that implementing a measure such a view flow used in the reviewer's suggested article would be useful in quantifying the activation of attention to past storms. Unfortunately, the lower threshold for n-gram usage rates in our dataset, combined with the fact that we've only stored the usage rate time series for the year after the storm occurs means our dataset is ill-suited to have a well defined flow rate between the vast majority of storms. We have added language discussing interactions between events in our future work section.

Comment: *The discussion on geography and the choice of language (English) ca be expanded.*

Response: We have added language on our choice to focus on English tweets and Hurricanes in the North Atlantic Basin.

Comment: *Moreover, I'd like to see a discussion on the build-up phase as opposed to post event attention.*

Response: We agree with the reviewer that the dynamics of build-up phase would be an interesting phenomenon to study, but feel this is beyond the scope of this paper. While the decay in attention paid to events is often modeled as an exponentially decaying pool in time, critically there is a constant initial attention at time zero. For Hurricanes, this assumption is justified because the damage potential of the storm is largely known by peak attention. The strength of the storm is widely reported, the locations effected are known, and initial estimates of the impacts are being shared.

For the build-up phase we must consider not only the time from the maximum future attention, but also changing estimates of the potential impact of the storm. There is a large uncertainty in both the trajectory and intensity of the still growing storm. Thus, we feel a model which assumes a constant peak attention would be unjustified.

Comment: *Finally, the authors might benefit from updating their literature review to include:*
Alshaabi, T., Minot, J. R., Arnold, M. V., Adams, J. L., Dewhurst, D. R., Reagan, A. J., ... & Dodds, P. S. (2020). How the world's collective attention is being paid to a pandemic: COVID-19 related 1-gram time series for 24 languages on Twitter. arXiv preprint arXiv:2003.12614.
Eom, Y. H., Puliga, M., Smailović, J., Mozetič, I., & Caldarelli, G. (2015). Twitter-based analysis of the dynamics of collective attention to political parties. PloS one, 10(7), e0131184.
Mitra, T., Wright, G., & Gilbert, E. (2017). Credibility and the dynamics of collective attention. Proceedings of the ACM on Human-Computer Interaction, 1(CSCW), 1-17.

Response: We thank the reviewer for these suggestions and have added references to these works.

Comment: *In L 79, L 83, and L 440 some cross references are missing.*

Response: The cross references have been amended.

Comment: *Additional response to 3. Have the authors made all data underlying the findings in their manuscript fully available?*
There is a link to a github page, but there is only a ReadMe file there. I could not easily locate the data as a separate file.

Response: The github page has been subsequently updated to include both data and code to reproduce the main figures.

Conclusion

We thank the reviewers and editor for their valuable feedback. We are pleased to resubmit our manuscript and look forward to seeing your response.

Sincerely and on behalf of the authors,

Michael V. Arnold
