# Supplementary material for: Hurricanes and hashtags: Characterizing online collective attention for natural disasters
Source: PLoS One. 2021 May 26;16(5):e0251762. doi: 10.1371/journal.pone.0251762 (PMC8153433; doi:10.1371/journal.pone.0251762)
Supplement: Attachment — Submitted filename: Hurricanes_Response_to_reviewers_2.docx [file pone.0251762.s003.docx]

Response to Reviewers

To the editors and reviewers,

Thank you again for the thorough and thoughtful reviews of our manuscript. We have read the new suggestions therein and have addressed the remaining concern in this letter. We discuss further our response to individual concerns below.

**Response to Academic Editor**

**Comment:** *The reviewers are happy with the revision. The only one thing Reviewer 1 wants to check is the usage of non-accented version of the word. The paper can be accepted if authors can quickly check this and provide a brief response.*

**Response:** We thank the editor and reviewers. We have examined the usage for both accented, and non-accented versions, and included some plots of our usage rate data. We believe our choice of the accented version is justified, and find that when restricting to only tweets classified as Spanish language, the correct accented version is used more frequency.

**Comment:** *We note that Figures F1, S14 - S21 in your submission contain map images which may be copyrighted. All PLOS content is published under the Creative Commons Attribution License (CC BY 4.0), which means that the manuscript, images, and Supporting Information files will be freely available online, and any third party is permitted to access, download, copy, distribute, and use these materials in any way, even commercially, with proper attribution. For these reasons, we cannot publish previously copyrighted maps or satellite images created using proprietary data, such as Google software (Google Maps, Street View, and Earth). For more information, see our copyright guidelines: http://journals.plos.org/plosone/s/licenses-and-copyright.*

*We require you to either (1) present written permission from the copyright holder to publish these figures specifically under the CC BY 4.0 license, or (2) remove the figures from your submission:*

**Response:** All maps submitted were generated using Basemap, an open source python library which allows unlimited use:

 https://github.com/matplotlib/basemap/blob/master/LICENSE

**Response to Reviewer #1**

***Comment:***  *I thank the authors for their replies to my points. There is only one thing that is not convincing me 100%. RE: spelling of "huracán", it's great to know that it was only a typesetting problem in the paper! However, I also pointed out that the accentless version of the hashtag (huracan) was more popular than either accented version. It's not super clear to me from the authors' reply that they checked also this version, which they probably should, given that it is a valuable quantity of data (although with an incorrect spelling). All in all, this should be possible to fix in a minor revision.*

**Response:** In response to the reviewer’s points, we have re-examined a few different accent scenarios. The reviewer suggested that the accent-less version of the hashtag is more popular than either accented version. We find this to be true for the hashtag of Hurricane Harvey. However, for Irma and Maria, we found the usage rates were almost the same. For the 2-gram version, the accented “Huracán María” is actually used more than “Huracan Maria”. In all cases the correctly accented 2-gram is used more( by an order of magnitude or two). Perhaps the reviewer was looking at hashtag data which does not separate hashtag popularity by tweet language first?

We agree with the reviewer that additional accented versions could be a valuable piece of data, but want to emphasize that all of the quantitative results in the main body of the paper are based only on English language tweets. We’ve included data from Spanish language 2-grams in Figure S8 of the supplementary materials to further investigate why Hurricane Maria’s measured attention is disproportionately low (relative to the destruction of the storm) in English tweets, and this figure helps to show that in Spanish tweets Maria receives comparable attention to other storms.

We appreciate the reviewer’s thoroughness, and hope to have addressed this concern.

**Response to Reviewer #2**

***Comment:*** *My comments and suggestions are fully addressed in the revision. The revised version is a much better paper than the initial submission and I'm glad that the authors were open to the suggestions. Best of luck!*

**Response:** Thank you again!

**Conclusion**

We thank the reviewers and editor for their valuable feedback. We are pleased to resubmit our manuscript and look forward to seeing your response.

Sincerely and on behalf of the authors,

Michael V. Arnold
